# Confocal laser endomicroscopy tears up the mask of protein-losing enteropathy

**DOI:** 10.1055/a-2155-8235

**Published:** 2023-09-15

**Authors:** Jie Xu, Lili Cai, Bota Cui

**Affiliations:** 1 Department of Microbiota Medicine and Medical Center for Digestive Diseases, The Second Affiliated Hospital of Nanjing Medical University, Jiangsu, China; 2 Key Lab of Holistic Integrative Enterology, Nanjing Medical University, Jiangsu, China; 3 Digestive Endoscopy and Medical Center for Digestive Diseases, The Second Affiliated Hospital of Nanjing Medical University, Jiangsu, China

A 35-year-old man was admitted to the hospital with a history of edema and hypoproteinemia for 27 months. Laboratory analysis revealed severe hypoalbuminemia, with a serum albumin level of 1.25 g/dL (normal range 4–5.5 g/dL). Other laboratory and imaging examinations revealed hypoproteinemia with no evidence of protein loss due to renal, hepatic, or autoimmune disease. Capsule endoscopy was performed to confirm there were no lesions in the small intestine and no lesions were visible under white-light endoscopy ( Fig. 1 ).

**Fig. 1:**
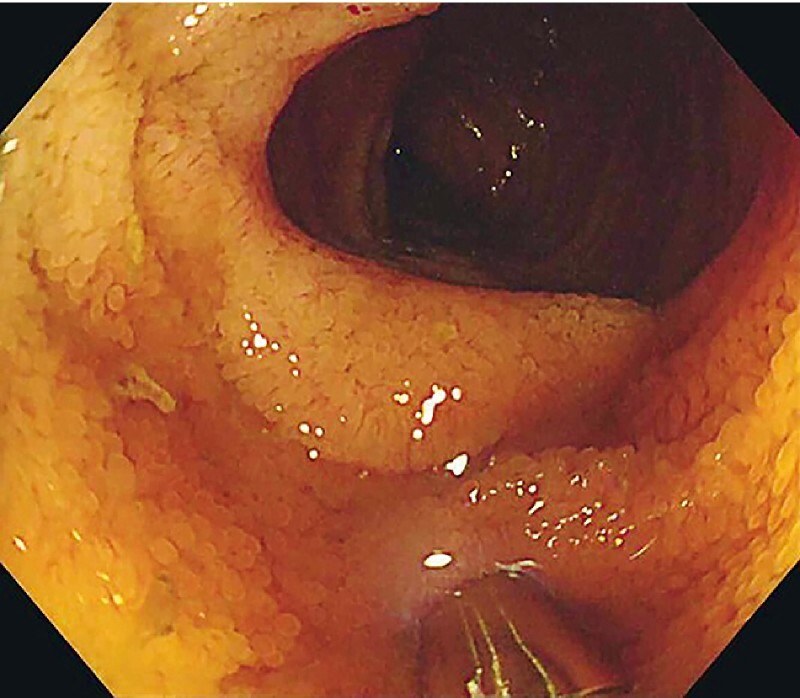
White-light endoscopic image showing the terminal ileum, with no obvious lesions visible.

A compromised epithelial barrier may lead to protein loss from the gut, but diagnosing lesions with a lack of significant subsurface mucosa is challenging. Therefore, after the patient had given written informed consent, we used probe-based confocal laser endomicroscopy (pCLE) to find clues with regard to albumin loss in the gut ( Video 1 ). The probe of the pCLE was inserted through the channel of the colonoscope and the colon and terminal ileum were reached during colonoscopy. The image from the pCLE revealed a noticeable flow of fluorescein from the epithelial cells in the terminal ileum ( Fig. 2 a ), indicating that the mucosal barrier was damaged. In contrast, the center of the colonic crypt opening remained dark ( Fig. 2 b ), indicating the colonic mucosal barrier was intact 1 . Given the combination of the clinical symptoms, examination outcomes, and the results of pCLE, the patient was diagnosed with protein-losing enteropathy (PLE), which refers to a condition that is characterized by hypoproteinemia and edema in the absence of proteinuria 2 .

**Fig. 2:**
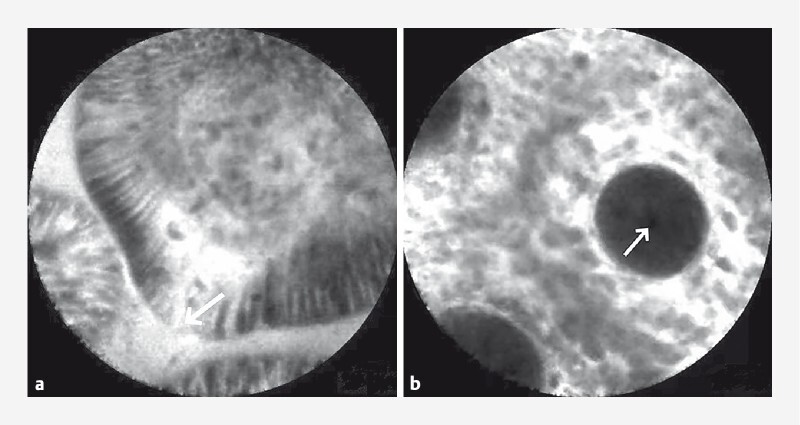
Images during probe-based confocal laser endomicroscopy (pCLE) showing: **a** in the terminal ileum, fluorescein flowing out of the epithelial cells (arrow); **b** in the colon, no leakage of fluorescein into the crypt lumen (arrow).

After receiving prednisone acetate combined with azathioprine, the patient’s anasarca improved and his serum albumin level was restored. He has reported no recurrence of his symptoms and continues to have a normal level of serum albumin.

This case indicates that pCLE would be the perfect tool to diagnose PLE, with the specific characteristics of dynamically detecting the real-time mucosal barrier function at the subcellular level.

**Video 1** Protein-losing enteropathy (PLE) is diagnosed via probe-based confocal laser endomicroscopy (pCLE) in a 35-year-old man.


Endoscopy_UCTN_Code_TTT_1AQ_2AI

## Notes

Endoscopy E-Videos

https://eref.thieme.de/e-videos
*E-Videos* is an open access online section of the journal *Endoscopy* , reporting on interesting cases and new techniques in gastroenterological endoscopy. All papers include a high-quality video and are published with a Creative Commons CC-BY license. Endoscopy E-Videos qualify for HINARI discounts and waivers and eligibility is automatically checked during the submission process. We grant 100% waivers to articles whose corresponding authors are based in Group A countries and 50% waivers to those who are based in Group B countries as classified by Research4Life (see: https://www.research4life.org/access/eligibility/ ). This section has its own submission website at https://mc.manuscriptcentral.com/e-videos
